# Gray Matter Volumetric Abnormalities Associated with the Onset of Psychosis

**DOI:** 10.3389/fpsyt.2012.00101

**Published:** 2012-12-03

**Authors:** Wi Hoon Jung, Stefan Borgwardt, Paolo Fusar-Poli, Jun Soo Kwon

**Affiliations:** ^1^Interdisciplinary Program in Neuroscience, Seoul National UniversitySeoul, South Korea; ^2^Institute of Human Behavioral Medicine, Seoul National University-MRCSeoul, South Korea; ^3^Department of Psychiatry, University of BaselBasel, Switzerland; ^4^Department of Psychosis Studies, Institute of Psychiatry, King’s CollegeLondon, UK; ^5^Department of Psychiatry and Behavioral Sciences, Seoul National University College of MedicineSeoul, South Korea; ^6^Brain and Cognitive Sciences-WCU program, College of Natural Sciences, Seoul National UniversitySeoul, South Korea

**Keywords:** psychosis, biomarker, gray matter, magnetic resonance imaging, schizophrenia, high-risk subjects

## Abstract

Patients with psychosis display structural brain abnormalities in multiple brain regions. The disorder is characterized by a putative prodromal period called ultra-high-risk (UHR) status, which precedes the onset of full-blown psychotic symptoms. Recent studies on psychosis have focused on this period. Neuroimaging studies of UHR individuals for psychosis have revealed that the structural brain changes observed during the established phases of the disorder are already evident prior to the onset of the illness. Moreover, certain brain regions show extremely dynamic changes during the transition to psychosis. These neurobiological features may be used as prognostic and predictive biomarkers for psychosis. With advances in neuroimaging techniques, neuroimaging studies focusing on gray matter abnormalities provide new insights into the pathophysiology of psychosis, as well as new treatment strategies. Some of these novel approaches involve antioxidants administration, because it is suggested that this treatment may delay the progression of UHR to a full-blown psychosis and prevent progressive structural changes. The present review includes an update on the most recent developments in early intervention strategies for psychosis and potential therapeutic treatments for schizophrenia. First, we provide the basic knowledge of the brain regions associated with structural abnormalities in individuals at UHR. Next, we discuss the feasibility on the use of magnetic resonance imaging (MRI)-biomarkers in clinical practice. Then, we describe potential etiopathological mechanisms underlying structural brain abnormalities in prodromal psychosis. Finally, we discuss the potentials and limitations related to neuroimaging studies in individuals at UHR.

## Introduction

Schizophrenia is the most disabling psychotic illness that affects approximately 1% of individuals worldwide. The disorder is characterized by a prodromal period that precedes the onset of full-blown psychotic symptoms, showing abnormalities in socialization and thinking (Yung and McGorry, 1996). Recent studies on psychosis have focused on the early course of the disorder (McGorry et al., 2008). The ultimate goal of researchers is to determine vulnerability- and disease-specific biological markers in an effort to predict the onset or course of the illness.

Over the past three decades, multiple neuroimaging studies have revealed brain abnormalities in patients with psychotic disorders. The notion that brain abnormalities contribute to the etiology of psychosis was initially proposed by Kraepelin (1919/1971) and Bleuler (1950) more than 110 years ago. This concept, however, was largely discredited during the twentieth century. A renewed interest in psychosis was generated after the first CT study by Johnstone et al. (1976). The first magnetic resonance imaging (MRI) study in patients with schizophrenia was published by Smith et al. (1984). Since then, the investigation of brain structure abnormalities associated with psychosis has flourished. MRI is the most established and used technique of for the investigation of the human brain structure *in vivo*. Over the past 27 years, MRI studies in patients with psychosis have played a pivotal role in providing support for the argument that brain abnormalities are associated with the disease. Moreover, this period has offered tremendous knowledge about the neurobiological basis of psychosis. Patients with psychosis have been shown to have enlarged lateral ventricles and reduced volume in the hippocampus, superior temporal gyrus (STG), and prefrontal cortices (PFC; Shenton et al., 2001; MacDonald and Schulz, 2009). Some discussion however was raised regarding the potential confounding role played by illness duration and antipsychotic treatment. Interestingly, some of these structural abnormalities are also observed in first-episode patients (FEP; Steen et al., 2006) who have a short duration of illness and a minimal exposure to antipsychotic treatment. Recently, new approaches that study individuals at high-risk for the disease have been used to better circumvent these problems and address this issue.

Specifically, two approaches focusing on high-risk subjects have been used to elucidate vulnerability to psychosis and minimizing confounding variables including neurodegenerative progress of the disease, institutionalization, and long-term treatment with antipsychotic medication. The genetic-high-risk (GHR) approach focuses on non-psychotic first-degree relatives of patients with schizophrenia, exploring trait markers of genetic vulnerability for the disorder, called endophenotypes. Alternatively, the clinical high-risk [CHR; also called ultra-high-risk (UHR), or at risk mental state (ARMS)] strategy focuses on individuals presenting with subthresholded psychotic features: (i) attenuated psychotic symptoms (APS), (ii) brief limited intermitted psychotic symptoms (BLIPS), and (iii) trait-plus-state risk factors [i.e., recent decline in social functioning and genetic risk for psychosis, genetic risk and deterioration syndrome (GRD; Yung et al., 2003]. Historically, the development of UHR criteria was introduced by Yung and McGorry, 1996; for a comprehensive state-of-the-art review on the high-risk state see, Fusar-Poli et al., 2012a). Research in the UHR field has progressed to the point that a new UHR diagnostic category is being considered for introduction in the forthcoming DSM-5 (Fusar-Poli and Yung, 2012). A recent meta-analysis in over than 2500 CHR subjects has showed that there is a consistent transition risk across different diagnostic criteria ranging from 18% at 6 months up to 36% after 3 years (Fusar-Poli et al., 2012b). The majority (73%) of these high-risk subject who will later transit to psychosis will develop a schizophrenic psychosis as compared to affective psychoses (11%, risk ratio 5.44; Fusar-Poli et al., 2012c). The high-risk state for psychosis is also associated with impaired quality of life and subtle impairments in cognitive functioning (Fusar-Poli et al., 2012d). In this scenario, neurobiological markers of liability to psychosis and transition to full illness can augment the predictive validity of current psychopathologically based criteria (Fusar-Poli and Broome, 2006; McGuire et al., 2008). To address this issue, neuroimaging studies of UHR patients may describe neurobiological markers of illness state as well as trait markers. However, despite the large number of structural imaging investigations in established psychosis and in UHR subjects, clinical applications for psychiatric neuroimaging are still lacking (Kloppel et al., 2012).

In the present review, recent MRI findings of UHR patients are critically reviewed along with multiple structural brain measures (regional brain volume and cortical thickness) suggesting that gray matter volume (GMV) abnormalities are associated with the onset of psychosis. Therefore, this review focuses on studies of MRI changes in UHR patients. The MRI studies discussed in this review are selected on a critical review of the available literature and they are listed in Table 1. The present article is not intended as a thorough review of all structural MRI studies in UHR patients [Wood et al., 2008; Pantelis et al., 2009; Jung et al., 2010; Smieskova et al., 2010; Borgwardt et al., 2011; Fusar-Poli et al., 2011a; Gogtay et al., 2011; for meta-analyses of structural findings in subjects at high clinical risk for psychosis (see Fusar-Poli et al., 2011b, 2012f) in subjects at genetic risk for psychosis (see Fusar-Poli et al., 2012e) for functional meta-analyses in subjects at clinical risk for psychosis (see Fusar-Poli et al., 2007; Fusar-Poli and Meyer-Lindenberg, 2012a,b)]. Rather, this review (i) provides basic knowledge of the brain regions associated with structural abnormalities in UHR patients; (ii) focuses on the use of MRI-biomarkers in clinical practice; (iii) suggests potential etiopathological mechanisms underlying structural brain abnormalities in prodromal psychosis; and (iv) discusses the potentials and limitations related to neuroimaging studies in individuals at UHR.

**Table 1:** **Neuroimaging studies investigating gray matter structures in ultra-high-risk subjects**.

Author (year)	Sample size	Conversion^†^ and follow-up^‡^	Measure	Main findings	Features/comments
**REGION OF INTEREST (ROI)**					
Phillips et al. (2002)	60 UHR	20 UHR-P	Hippocampus, whole-brain volumes	HC > UHR: bilateral hippocampus	The first ROI study of UHR reporting GMV abnormalities in hippocampus
	32 FEP	40 UHR-NP		UHR-P > UHR-NP or FEP: L. hippocampus	
	139 HC			HC > UHR-NP: R. hippocampus	
				UHR-P vs. HC: no significant differences	
Yücel et al. (2003)	63 UHR	21 UHR-P	ACC morphology	UHR: more interrupted L cingulate sulcus and poorly developed paracingulate sulcus	The first study to examine the surface morphology of ACC in UHR using a reliable method of capturing the sulcal and gyral variation in ACC
	75 HC	42 UHR-NP		UHR-NP vs. UHR-P: no significant differences	
Garner et al. (2005)	94 UHR	31 UHR-P	Pituitary volume	UHR-P > UHR-NP: increased pituitary volume	The first study of pituitary volume in UHR as an indirect measure of hormonal stress response and HPA-axis activity
	49 HC	63 UHR-NP		HC vs. UHR: not reported	
Wood et al. (2005)	79 UHR (35 UHR-FH+ 44 UHR-FH−)	24 UHR-P	Hippocampus volume	UHR-FH+ > UHR-FH−: L hippocampal volume	The study to investigate the effect of family history in hippocampal volumes and ACC morphology
	49 HC		ACC morphology	UHR-FH+ and UHR-FH−: similar pattern of L ACC and trend level difference of reduced PCS folding and more frequent CS interruptions	A family history of psychosis is not associated with a greater degree of structural brain abnormalities in UHR
Velakoulis et al. (2006)	135 UHR	39 UHR-P	Hippocampus, amygdala and whole-brain volumes	UHR: whole-brain volume reduction	The study to investigate hippocampal and amygdala volumes in a large sample of patients with chronic SZ, FEP, UHR, and HC
	89 SZ 162 FEP	96 UHR-NP		UHR-NP vs. UHR-P: no differences in hippocampus and amygdala volumes	
	87 HC	
Thompson et al. (2007)	23 UHR		Pituitary and hippocampus volume, whole-brain volume	No correlations volumes with plasma cortisol level or glucocorticoid receptor numbers	The study to examine the relationship between the experience of stressful events, HPA-axis functioning (plasma cortisol levels), hippocampal and pituitary volumes and psychotic symptoms in UHR
Hurlemann et al. (2008)	36 UHR (20 EPS 16 LPS)	8 UHR-P (3 EPS 5 LPS)	Hippocampus volume	EPS and LPS: bilateral reduced hippocampal volumes	The study to investigate the relationship between hippocampal volume and function (verbal learning) in UHR
	36 HC		Rey auditory verbal learning test	
Takahashi et al. (2009b)*	97 UHR	31 UHR-P	Insular volume	In cross-sectional comparison: UHR-NP > UHR-P: insular volumes bilaterally HC > UHR-P: R insular volumes	The first ROI study to report gray matter changes of insular subregions in UHR in both cross-sectional and longitudinal designs.
	55 HC	66 UHR-NP 51 rescanned^‡^ (11 UHR-P 20 UHR-NP 20 HC)		In longitudinal comparison: UHR-NP and HC > UHR-P: bilateral insular volumes	
Takahashi et al. (2009a)*	35 UHR	12 UHR-P	STG and its subregion volumes	In cross-sectional comparison: HC > UHR-P: planum temporal at follow-up	The first ROI study to report progressive gray matter reduction of STG subregions during prodromal phase and after the onset of psychosis in UHR
	23 FEP 22 HC	23 UHR-NP rescanned^‡^ (all subjects)		In longitudinal comparison: HC and UHR-NP > UHR-P and FEP: planum polare, planum temporal, and caudal region		
Buehlmann et al. (2010)	37 UHR	16 UHR-P	Hippocampus volume	UHR and HC > FEP: L hippocampus	The study to investigate hippocampal volume according to psychosis stage (i.e., FEP, UHR, HC)
	23 FEP	21 UHR-NP		UHR-P > FEP but no HC: L hippocampus volume	
	22 HC			UHR-P, UHR-NP, FEP, and HC: no significant trend in left hippocampus	
				UHR-P vs. UHR-NP: no significant differences	
Witthaus et al. (2010)	29 UHR	8 UHR-P	Hippocampus and amygdala volumes	HC > UHR: bilateral hippocampus corpus and tail	The study to examine GMV in hippocampal subregions and amygdala
	23 FEP			UHR-NP > UHR-P: R hippocampus corpus and tail	
	29 HC			UHR and HC > FEP: L amygdala volumes	
Takahashi et al. (2010)	97 UHR	31 UHR-P	STG and its subregion volumes	HC > UHR: bilateral STG at baseline	The study to investigate GMV in STG subregions in antipsychotic-naïve UHR
	42 HC	66 UHR-NP		UHR-P vs. UHR-NP: no significant differences	
Wood et al. (2010)	66 UHR	7 UHR-P	Hippocampus volume, T2 relaxation time, and MRS	HC > UHR-P or UHR-NP: L hippocampal volumes	The first study to combine multiple modalities imaging to investigate the integrity of the hippocampus and the first to examine T2 relaxometry in UHR
	29 HC	59 UHR-NP		HC > UHR-NP: R hippocampal volume	
Hannan et al. (2010)	78 UHR (32 UHR-FH+, 29 UHR-FH−)	39 UHR-P	Caudate nucleus volume, neurocognitive measures	UHR vs. HC: no significant differences	The study to investigate GMV in caudate nuclei and the relationship to negative symptoms and neurocognitive performance on a task of spatial working memory in neuroleptic-naïve UHR
	39 HC	39 UHR-NP		UHR-NP vs. UHR-P: no significant differences	
				UHR-FH + vs. UHR-FH-: no significant differences	
				UHR-P: correlations between executive function and CNV	
Bechdolf et al. (2012b)	22 UHR	11 UHR-BP	Amygdale, lateral ventricles, insula, hippocampus	UHR-BP and UHR-NP > HC: hippocampus volume	The first study to neuroanatomically investigate young people with bipolar disorder prior to the onset of first-episode mania
	11 HC	11 UHR-NP		HC > UHR-BP: amygdala volume	
		11 HC		HC > UHR-BP: insular volume	
**VOXEL-BASED MORPHOMETRY (VBM)**					
Pantelis et al. (2003)*	75 UHR	23 UHR-P	VBM	In cross-sectional comparison: UHR-NP > UHR-P: R medial and lateral temporal and inferior FG, and bilateral cingulate cortex	The first VBM study to report gray matter changes in UHR in both cross-sectional and longitudinal designs.
		52 UHR-NP		In longitudinal comparison: UHR-P: longitudinal reduction in L parahippocampal, fusiform, cingulate and cerebellar cortices, and OFC; UHR-NP: longitudinal reduction in L cerebellum
		21 rescanned^‡^ (10 UHR-P 11 UHR-NP)			
Borgwardt et al. (2007a)	35 UHR	12 UHR-P	VBM-SPM2	UHR vs. FEP vs. HC: difference in L insula, STG, ACC, and precuneus	The study to clarify the nature of neuroanatomical abnormalities in UHR by comparing them with both controls and FEP
	25 FEP	23 UHR-NP		HC > UHR: L medial temporal cortex	
	22 HC			UHR-NP > UHR-P: R insula, inferior FG, and STG	
Borgwardt et al. (2007b)	12 UHR-P		VBM-SPM2	HC > UHR-P: bilateral PCC, precuneus, paracentral lobule, and L superior parietal lobule and greater L parietal/posterior temporal region	The study to assess regional GMV in only UHR-P to reduce heterogeneity in UHR (i.e., UHR-P, UHR-NP) by comparing them with both controls and FEP
	25 FEP 22 HC			FEP > UHR-P: bilateral temporal gyrus, R lentiform nucleus volumes	
Meisenzahl et al. (2008)	40 UHR	15 UHR-P	VBM-SPM5	HC > UHR: frontal, lateral temporal and medial temporal regions	The study to investigate GMV in UHR using the new unified segmentation model of SPM5
	75 HC	25 UHR-NP		Inverse correlations between prefrontal gray matter volume and PANSS scores	
Borgwardt et al. (2008)*	20 UHR	20 rescanned^‡^ (10 UHR-P 10 UHR-NP)	VBM-SPM5	UHR-P: longitudinal volume reductions in OFC, superior frontal, inferior temporal, medial and superior parietal cortex, and cerebellum	One of the first studies to show progressive GMV changes in UHR-P
				UHR-NP: No longitudinal changes	
Ziermans et al. (2009)	54 UHR	7 UHR-P	VBM	HC vs. UHR: no significant differences	The study to investigate GMV in young UHR sample of adolescents aged 12-18 years
	54 HC				None of the measures differed between UHR and HC
Koutsouleris et al. (2009a)	46 UHR (20 EPS, 26 LPS)	15 UHR-P (1 EPS, 14 LPS)	VBM-SPM5	HC > LPS: frontotemporo limbic structures in LPS	The first study that characterized structural brain abnormalities in UHR sample selected for basic symptoms
	75 HC	18 UHR-NP		HC > EPS: bilateral temporolimbic alterations and subtle prefrontal abnormalities	
				UHR-P vs. UHR-NP and HC: prefrontal abnormalities in UHR-P	
Witthaus et al. (2009)	30 UHR	1 UHR-P	VBM-SPM2	HC > UHR: bilateral cingulate gyrus and hippocampus, R inferior frontal and STG	The study to investigate GMV abnormalities according to psychosis stage
	23 FEP 29 HC			FEP > UHR: bilateral cingulate cortex and hippocampus, L parahippocampus, OFC, amygdala and fusiform gyrus, R STG, inferior frontal and temporal pole	
Smieskova et al. (2012b)	31 UHR (18 ARMS-ST, 13 ARMS-LT)		VBM-SPM8	ARMS-LT and FEP > ARMS-ST: bilateral insular volumes	The study to investigate GMV abnormalities associated with transition probability and the reduced risk of developing psychosis by comparing four neuroleptic-free groups (i.e., FEP, ARMS-ST, ARMS-LT, HC)
	16 FEP			ARMS-ST > FEP: L parahippocampal gyrus	
	19 HC			ARMS-LT > FEP: L parahippocampal gyrus	
				ARMS-LT > HC: R insula	
				Whole ARMS: correlations between gray matter volume and global functioning and positive/negative symptoms	
Dazzan et al. (2012)	102 UHR	28 UHR-P (19 UHR-SZ 7 UHR-AFF)	VBM-SPM5	UHR-NP > UHR-SZ: bilateral postcentral gyrus, R middle temporal gyrus, L pars opercularis	The first VBM study to perform an exploratory analysis of the specificity of GMV changes in UHR to later schizophreniform and affective psychosis
		74 UHR-NP		UHR-NP > UHR-AFF: R superior FG	
				UHR-SZ > UHR-AFF: subcallosal cingulate gyrus	
Whitford et al. (2012)	25 UHR-HSV1+ 33 UHR-HSV1- 19 HC		VBM-SPM2	HSV1+ vs. HSV1− vs. HC: no significant differences In ROI analysis: HSV1− or HC > HSV1+: cuneus	The first study that has identified cuneus abnormalities in UHR individuals with a history of HSV1 infection relative to HC
**CORTICAL SURFACE ANALYSIS**					
Fornito et al. (2008)	70 UHR	35 UHR-P	Surface-based ACC morphometry	HC > UHR-P: thickness of a rostral paralimbic ACC region UHR-NP > HC: thickness in dorsal and rostral limbic areas	The study to investigate ACC morphometry applying a cortical surface-based protocol for parcellating the ACC in large sample of UHR
	33 HC (from two sites)	35 UHR-NP			
Sun et al. (2009a)*	35 UHR	35 rescanned^‡^ (12 UHR-P 23 UHR-NP)	Cortical pattern matching	UHR-P > UHR-NP: Greater brain surface contraction in R prefrontal region UHR-P: non-significant trend in L prefrontal region and bilateral occipital region	The first study using cortical pattern matching to compare longitudinal brain surface contractions in UHR-P and UHR-NP
Jung et al. (2011)	29 EPS	8 UHR-P	Surface-based cortical thickness (CLASP)	HC > UHR: STG, MTG, PFC, parietal cortex, ACC, parahippocampal cortex	The first study to investigate cortical thickness abnormalities according to psychosis stage
	29 HC	21 UHR-NP			
	31 SZ				
Ziermans et al. (2012)*	43 UHR	8 UHR-P	Surface-based cortical thickness (CLASP), brain volumes, VBM	In cross-sectional comparison: No group differences	The first study to investigate progressive changes in both GMV and cortical thickness in UHR
	30 HC	35 UHR-NP rescanned^‡^ (all subjects)		In longitudinal comparison: HC > UHR: L middle temporal gyrus; HC > UHR-P: L ACC, precuneus, temporo-parieto-occipital area; HC vs. UHR-NP: no differences	
**MULTIMODAL STUDY**					
Fusar-Poli et al. (2011c)*	15 UHR	2 UHR-P	VBM-SPM5, fMRI; N-back task	In cross-sectional comparison: HC > UHR: middle and medial FG, insula, ACC; UHR: Positive interaction between BOLD and middle FG volume	The first study to longitudinally employ fMRI and VBM in UHR
	15 HC	30 rescanned^‡^ (15 UHR 15 HC)		In longitudinal comparison: UHR: no longitudinal changes	
Fusar-Poli et al. (2011d)*	39 UHR	10 UHR-P	VBM-SPM5, ERP; auditory oddball paradigm	In cross-sectional comparison: HC > UHR: R superior FG, R supramarginal/superior temporal gyrus, L medial FG, L inferior FG, R orbital gyrus; UHR-NP > UHR-P: R inferior parietal lobule, L parahippocampal gryus	The first study to report that a reduction in P300 amplitude is related to GMV loss in UHR
	41 HC	29 UHR-NP		In longitudinal comparison: UHR: decrease in bilateral superior FG, R caudate but increase in R inferior FG, R ACC, L cerebellum; UHR-NP: decrease in bilateral superior FG, R caudate, L putamen; UHR-P: decrease in R middle temporal gyrus
		36 rescanned^‡^ (5 UHR-P 17 UHR-NP 14 HC)			
Smieskova et al. (2012c)	17 ARMS-ST		VBM-SPM8, fMRI; N-back task	HC > FEP: anterior cingulo-prefrontal, hippocampal, occipito-cerebellar regions	The study to investigate functional and structural brain differences between individuals with vulnerability to develop psychosis, particularly higher vs. lower probability of developing psychosis (ARMS-ST, ARMS-LT), and FEP neurofunctional differences within ARMS were related to different duration of ARMS and these abnormalities were directly related to volumetric reduction
	16 ARMS-LT			ARMS-LT > FEP: temporo-insula	
	21 FEP			ARMS-ST > FEP: fronto-parietal and occipital regions	
	20 HC			HC > ARMS-ST and ARMS-LT: ACC, frontal volumes	
				ARMS-LT > ARMS-ST: temporal gyrus extending to insula	
				ARMS-LT: the association between reduced insular and PFC activation and GMV reduction in the same area	
Stone et al. (2009)	27 UHR		VBM-SPM5, MRS	HC > UHR: orbitofrontal gyrus, ventral ACC	The first study to examine the relationship between glutamate levels and GMV in UHR
	27 HC			UHR > HC: L cerebellum, L occipital cortex	
				UHR: a correlation between thalamic glutamate level and GMV in medial temporal and insula	
**META-ANALYSIS**					
Smieskova et al. (2010)	385 HR 211 FEP	95 HR-P	Effect size Cohen’s d	HR-NP > HR-P: Small to medium effect sizes of prefrontal, cingulate, insular, and cerebellar GMV	The study to investigate structural and functional brain abnormalities in UHR-P in relation to UHR-NP, HC, and FEP applying meta-analysis
	290 HC	
Fusar-Poli et al. (2011b)^a^	879 HR (320 UHR, 559 GHR)	Unclear	Voxel-based meta-analysis using ALE	HC > HR: STG, parahippocampal gyrus, precuneus, medial FG, ACC	The study to investigate GMV abnormalities in HR by performing meta-analysis of only whole-brain VBM studies using ALE technique
	708 HC			HC > UHR: parahippocampal gyrus, STG, insula, inferior FG, medial FC	
				HR-NP > HR-P: inferior FG, STG	
				HC > GHR: parahipoocampal gyrus, ACC/GHR > HC: ACC	
				UHR > GHR: parahippocampal gyrus, insula	
				HR > SZ: parahippocampal gyrus, medial FG, middle temporal gyrus, precuneus	
Fusar-Poli et al. (2012f)	198 UHR		Voxel-based meta-analysis using SDM	HC > UHR: middle/superior temporal gyrus, parahippocampal gyrus, ACC, middle FG	The largest whole-brain structural meta-analysis examining GM abnormalities in antipsychotic-naïve subjects in relation to psychosis onset
	206 FEP			HC > FEP: STG, insula, cerebellum	
	202 HC			HR > FEP: cingulate, cerebellum, STG, insula	
**PREDICTION OF DISEASE CLASSES USING CORTICAL GRAY MATTER DIFFERENCES**					
Haller et al. (2009)	20 UHR		Cortical thickness analysis, cortical thickness asymmetry	No difference in direct cortical thickness	The study to report that the intraindividual analysis of cortical thickness asymmetry helped discriminate between groups
	20 FEP 20 HC			Cortical thickness asymmetry in frontal, temporal and parietal regions help distinguish between UHR and HC	
Koutsouleris et al. (2009b)	20 EPS	15 UHR-P	VBM-SPM5, RAVENS maps, SVM	Classification accuracies: (i) HC vs. the rest (86%), EPS vs. the rest (91%), LPS vs. the rest (86%); (ii) HC vs. the rest (90%), UHR-P vs. the rest (88%), UHR-NP vs. the rest (86%)	The first study to evaluate the feasibility of early recognition and disease prediction in UHR using multivariate neuroanatomical pattern classification
	25 LPS 25 HC	18 UHR-NP		Neuroanatomical mapping of SVM decision function: UHR-P vs. HC and UHR-P vs. HC: ACC, PCC, OFC, LPFC, LTG, medial TG, caudate; UHR-P vs. UHR-NP: medial and LTG, LPFC, thalamus, cerebellum
Koutsouleris et al. (2012)	37 UHR	16 UHR-P	VBM-SPM5, RAVENS maps, SVM	Classification accuracies: HC vs. UHR-P (92.3%), HC vs, UHR-NP (66.99%), UHR-P vs. UHR-NP (84.3%)	The replication study to confirm their previous findings (75) and to support that the early prediction of psychosis may be reliably enhanced using neuroanatomical pattern recognition operating at the single-subject level
	22 HC	21 UHR-NP		Neuroanatomical mapping of SVM decision function: HC VS. ARMS-T: PFC, insula, medial and lateral parietal cortex, basal ganglia, thalamus, cerebellum; HC vs. ARMS-NT: ACC, PCC, inferior temporal and fusiform cortices; AMRS-T vs. ARMS-NT: medial FG, precuneus, premotor area, dorsolateral FC, parahippocampal and inferior temporal cortex, thalamus	
Koutsouleris et al. (2010a)*	25 UHR	53 rescanned^‡^ (25 UHR, 28 HC)	VBM-SPM5 (DBM), PLS, SVR	UHR > HC, UHR-P > UHR-NP: pronounced morphometric changes in L PFC, perisylvian, parietal, periventricular areas	The first study to investigate vulnerability and disease-related brain trajectories in UHR using machine-learning techniques
	28 HC			A reliable prediction of longitudinal brain changes using SVR: HC vs. UHR (*r* = 0.83), HC vs. UHR-P vs. UHR-NP (*r* = 0.83)	
**MULTICENTER STUDY**					
Mechelli et al. (2011)	182 UHR	48 UHR-P	VBM-SPM8	HC > UHR: medial orbital gyrus, bilateral gyrus rectus, R ACC	The first multicenter VBM study of UHR individuals for psychosis
	167 HC (from five sites)	134 UHR-NP		UHR-NP > UHR-P: L parahippocampal gyrus	
**BRAIN-COGNITION ASSOCIATIONS**					
Koutsouleris et al. (2010b)	40 UHR	Among 27 UHR	VBM-SPM8, TMT-B task	HC: correlation between insula and TMT-B	The first study to report on neuroanatomical correlates of executive impairment in UHR
	30 HC	11 UHR-P		UHR: correlation between ventromedial PFC GMV (negatively) and cerebellum (positively) and TMT-B	
		16 UHR-NP			

*UHR, ultra-high-risk subjects; FEP, first-episodic patients; HC, healthy controls; SZ, schizophrenia patients; GHR, genetic-high-risk subjects; UHR-P, those who convert to psychosis; UHR-NP, those who did not convert to psychosis; UHR-FH+, UHR subjects with family history of psychosis; UHR-FH-, UHR subjects without family history of psychosis; EPS, UHR subjects in early prodromal states; LPS, UHR subjects in late prodromal states; UHR-BP, UHR who converted to a diagnosis of bipolar I or II disorder; UHR-SZ, UHR who converted to a diagnosis of schizophrenia; UHR-AFF, UHR who converted to a diagnosis of affective psychosis; ARMS, individuals with an at risk mental state (=UHR); ARMS-ST, short-term UHR since first presentation; ARMS-LT, long-term UHR since first presentation; UHR-HSV1+, UHR individuals with a history of Herpes Simplex Virus type 1 exposure; UHR-HSV1−, UHR individuals without a history of Herpes Simplex Virus type 1 exposure; L, left; R, right; PANSS, the *Positive and Negative* Syndrome Scale; VBM, voxel-based morphometry; SPM, Statistical Parametric Mapping software; CLASP, Constrained Laplacian Anatomic Segmentation using Proximity; fMRI, functional magnetic resonance imaging; ERP, event-related potentials; MRS, magnetic resonance spectroscopy; ALE, activation likelihood estimation techinique; SDM, signed differential mapping; RAVENS, Regional Analysis of Volumes in Normalized Space; DBM, deformation-based morphometry; PLS, partial-least-squares; SVM, support vector machine; SVR, support vector regression; TMT, trail making test; ACC, anterior cingulate cortex; AI, adhesion interthalamica; CSP, cavum septum pellucidum; CS, cingulate sulcus; CNV, caudate nucleus volume; FG, frontal gyrus; GM, gray matter; GMV, gray matter volume; MTG, middle temporal gyrus; OFC, orbitofrontal cortex; PCS, paracingulate sulcus; PCC, posterior cingulate cortex; PFC, prefrontal cortex; STG, superior temporal gyrus; HPA-axis, hypothalamic-pituitary-adrenal axis*.

*Indicates longitudinal MRI study.

*^†^Indicates psychosis conversion rate during clinical follow-up*.

*^‡^Indicates sample size involved in MR follow-up*.

*^a^The number of subjects evaluated from Table 1 inserted in the paper*.

## GM Abnormalities in Individuals at UHR

### Cross-sectional comparison between individuals at UHR and healthy controls

Magnetic resonance imaging studies of patients with established psychosis have revealed brain structural abnormalities associated with the disorder. Individuals at UHR, however, may demonstrate some specific markers of potential vulnerability to psychosis. Accumulating data has revealed that neurobiological abnormalities in UHR subjects are qualitatively similar to but less severe than are those in patients with established psychosis.

Previous studies employing region of interest (ROI) or whole-brain voxel-based morphometric (VBM) methods have reported reduced hippocampal GMV in UHR individuals compared with healthy controls (Phillips et al., 2002; Wood et al., 2005, 2010; Borgwardt et al., 2007a; Hurlemann et al., 2008; Buehlmann et al., 2010; Witthaus et al., 2010), although results have been inconsistent (Velakoulis et al., 2006). Studies have shown that UHR individuals displayed deficits in hippocampal-associated verbal memory (Hurlemann et al., 2008). Wood et al. (2005) reported decreased hippocampal volume in UHR patients without a family history of psychosis compared with individuals with a family history of psychosis, suggesting that non-specific environmental factors are more highly associated with morphological abnormalities than are genetic factors.

Prefrontal cortex abnormalities in UHR patients have been reported in the lateral PFC (Meisenzahl et al., 2008; Koutsouleris et al., 2009a; Witthaus et al., 2009), medial PFC, and ACC (Borgwardt et al., 2007a; Witthaus et al., 2009; Jung et al., 2011; Choi et al., 2012). Moreover, using VBM and functional MRI (fMRI) techniques, reduced GMV in the middle frontal gyrus was correlated with reduced neural activity in this region (Fusar-Poli et al., 2011c). Deficits in the ACC may provide evidence of early neurodevelopmental anomalies in schizophrenia, as reports have indicated alterations in the pattern of cortical folding in this region (Yücel et al., 2003). Altered cortical folding patterns in UHR individuals without a family history of psychosis, compared with individuals with a family history, suggested involvement of environmental rather than genetic factors (Wood et al., 2005). Specifically, abnormalities in the midline cortical structures (i.e., medial PFC and ACC) in UHR patients may contribute to their disrupted sense of self (Nelson et al., 2009). Recently, UHR individuals were shown to have aberrant connectivity in the default mode network, which is involved in self-referential processing (Shim et al., 2010). Reduced GMV in the parietal area in UHR patients was also observed (Fusar-Poli et al., 2011d), and cortical thinning in the parietal regions in UHR patients was reported (Jung et al., 2011). However, Haller et al. (2009) reported no differences in cortical thickness, but did find differences in cortical asymmetry in the parietal, insular, and occipitotemporal gyri between UHR individuals and controls. A recent magnetoencephalography (MEG) study in UHR patients showed aberrant alpha modulation related to selective attention, particularly in the parieto-occipital region (Koh et al., 2011). A multimodal EEG-MRI study reported a relationship between P300 amplitude and GMV in the supramarginal gyrus in UHR patients (Fusar-Poli et al., 2011d).

Volumetric abnormalities in the lateral temporal lobe, particularly the STG, in UHR patients have frequently been reported. The STG processes sounds and social information and is related to functional deficits, including auditory hallucinations and thought disorders (Allen, 2008; Sun et al., 2009b). Several ROI and VBM studies in UHR patients have reported reduced STG volume (Borgwardt et al., 2007a; Koutsouleris et al., 2009a; Witthaus et al., 2009; Takahashi et al., 2010), although these findings are inconsistent (Takahashi et al., 2009a). Recent studies of surface-based cortical thickness were consistent with previous findings and indicated reduced STG volume in UHR patients (Jung et al., 2011). MEG studies have identified aberrant auditory processing in UHR individuals, including defects in N1m adaptation, the magnetic counterpart of MMN (MMNm), and correlations between MMNm and clinical symptoms (Shin et al., 2009, 2012).

Aberrant GMV in limbic system regions such as the insula and amygdala has also been reported. UHR individuals showed reduced insular volume (Borgwardt et al., 2007a,b; Takahashi et al., 2009b) but no differences in amygdala volume (Velakoulis et al., 2006; Witthaus et al., 2009). A recent study investigated the volume of the caudate nuclei using ROI techniques (Hannan et al., 2010). Caudate nuclei volumes did not differ significantly between UHR individuals and healthy controls.

Given that the medial PFC, ACC, inferior frontal gyrus, insula, and STG are involved in social cognitive processes (Blakemore, 2008), UHR individuals may display abnormal social functioning. There is significant evidence to support this hypothesis [i.e., impaired social functioning (Shim et al., 2008) and social cognition (Chung et al., 2008)]. Patients with psychosis have alterations in facial processing (Shin et al., 2008). Interestingly, UHR individuals also display similar deficits (Kim et al., 2010), suggesting a role for social dysfunction in psychosis.

### Cross-sectional comparison between individuals at UHR-P and UHR-NP

Individuals at UHR are a heterogeneous group that includes individuals who later develop psychosis (UHR-P) and those who do not (UHR-NP). Some brain abnormalities may reflect an underlying vulnerability for psychosis, whereas others may be associated with progression due to an acute illness. Therefore, differences in GMV between individuals at UHR-P and UHR-NP are of specific interest.

In ROI studies, individuals at UHR-P displayed smaller insular (Takahashi et al., 2009b) and hippocampal GMV (Wood et al., 2010) and increased pituitary volume (Garner et al., 2005). Phillips et al. (2002) reported greater hippocampal volume in UHR-P patients compared with UHR-NP patients and FEP. No GMV differences were noted between UHR-P and UHR-NP in several brain regions including the STG (Takahashi et al., 2010), hippocampus (Buehlmann et al., 2010), amygdala (Velakoulis et al., 2006), caudate nucleus (Hannan et al., 2010), or in ACC cortical folding patterns (Yücel et al., 2003). However, the caudate nucleus volume in UHR-P patients was positively correlated with total errors in spatial working memory and verbal fluency performance (Hannan et al., 2010). In VBM studies, UHR-P patients displayed smaller GMV in the insula and in the ACC compared with UHR-NP patients (Pantelis et al., 2003; Borgwardt et al., 2007a). Borgwardt et al. (2007b) found no differences in these two regions between individuals at UHR-P and FEP. Reductions in the PFC and orbitofrontal cortex, however, were noted (Koutsouleris et al., 2009a; Dazzan et al., 2012). Fornito et al. (2008) assessed cortical thickness in the ACC in UHR patients and found that UHR-P individuals showed cortical thinning in the rostral paralimbic region. A recent study reported decreased cortical thickness in the ACC, PFC, STG, and inferior temporal cortex in UHR-P individuals (Jung et al., 2011). Moreover, UHR-P individuals displayed poor social cognition (Kim et al., 2011) and social functioning compared with UHR-NP individuals (Jang et al., 2011).

### Longitudinal comparison

Longitudinal studies have focused on the pathophysiological nature of brain abnormalities around the time of the onset of illness. Using the VBM approach, Pantelis et al. (2003) reported that UHR-P patients displayed longitudinal reductions in the orbitofrontal, cerebellar, fusiform, and parahippocampal cortices and the cingulate gyrus. Borgwardt et al. (2008) reported progressive volume reductions in the orbitofrontal cortex and cerebellum, as well as the superior frontal, inferior temporal, and superior parietal cortices and the precuneus. ROI studies have found greater reductions in the insula (Takahashi et al., 2009b) and STG (Takahashi et al., 2009a) in UHR-P patients compared with patients at UHR-NP. Using cortical pattern matching techniques, Sun et al. (2009a) revealed greater brain contraction in the PFC in UHR-P patients compared with that in UHR-NP patients. A recent surface-based cortical thickness study found that UHR-P individuals displayed cortical thinning in widespread regions in the ACC, precuneus, and temporo–parieto–occipital areas (Ziermans et al., 2012). Recent longitudinal multimodal studies have used MRI with other neuroimaging modalities. A longitudinal electroencephalogram (EEG)-VBM study reported progressive GM alterations in the PFC and subcortical areas, including the caudate, in patients at UHR, but no significant changes over time in the P300 amplitude during a task (Fusar-Poli et al., 2011d). UHR-P patients showed reduced GMV in the middle temporal gyrus, and UHR-NP patients showed decreased GMV in the superior frontal gyrus, caudate, and putamen between a baseline and follow-up session. In contrast, no significant differences in GMV were noted between the baseline and the follow-up scan in UHR patients in another longitudinal fMRI-VBM study from the same center (Fusar-Poli et al., 2011c). However, longitudinal increases in activation in the parahippocampus and ACC were noted. Taken together, these findings suggest that GMV abnormalities in some brain regions dynamically emerge during the acute process of transition to psychosis, particularly showing progressive changes in the frontal and temporal regions in UHR-P patients.

### Voxel-wise meta-analyses and multicenter studies

There is accumulating evidence for structural changes in UHR individuals. Moreover, researchers have recently conducted voxel-wise meta-analysis studies in UHR patients (Smieskova et al., 2010; Fusar-Poli et al., 2011b, 2012f). Smieskova et al. (2010) reported decreased PFC, cingulate, insula, and cerebellar GMV in UHR-P patients compared with UHR-NP patients. Methodological differences and different high-risk statuses (i.e., GHR, UHR) were noted between the studies. However, changes in the PFC, ACC, medial temporal, and cerebellar cortex may predict the development of psychosis in UHR patients. To address the heterogeneity across studies that use different methods, Fusar-Poli et al. (2011b) conducted a voxel-wise meta-analysis of whole-brain VBM studies in high-risk individuals including UHR or GHR. UHR patients compared with healthy controls displayed reduced GMV in the STG, parahippocampal region, precuneus, medial and middle frontal gyri, and the ACC. Compared with UHR-NP patients, UHR-P patients showed baseline GM reductions in the inferior frontal gyrus and the STG. UHR individuals relative to GHR displayed greater volume in the parahippocampal gyrus, insula, and STG and less volume in the ACC. Additionally, the study confirmed significant effects of atypical antipsychotic treatment on the inferior frontal gyrus and insula. To remove confounding effects of antipsychotic drugs, Fusar-Poli et al. (2012f) conducted an additional voxel-wise meta-analysis on VBM studies enrolling antipsychotic-naïve subjects. GMV reductions were found in the middle and superior temporal gyri, hippocampal regions, and middle frontal gyrus in patients at UHR, and in the STG, insula, and cerebellum in FEP patients. Compared with UHR subjects, FEP subjects showed GM decreases in the STG, ACC, insula, and cerebellum. Moreover, an inverse correlation was noted between STG GMV and symptom severity, suggesting that the STG is associated with the onset of psychotic symptoms.

A multicenter study conducted on UHR patients collected data from five sites (subjects were 167 healthy controls, 48 UHR-P, 134 UHR-NP) and was recently published (Mechelli et al., 2011). The UHR group had less GMV in the frontal regions compared with controls. Compared with patients with UHR-NP, UHR-P patients had less GMV in the parahippocampal cortex.

## The Feasibility of Neuroanatomical Biomarkers

Despite the convincing evidence from neuroimaging studies, the feasibility of using MRI-based data on structural changes remains far in the future for clinical practice in psychiatry. This is largely due to the overlap with anatomical variation within the normal range. Recently, advancements in computer visualization and neuroimaging techniques have raised intense interest in diagnostic MRI-based biomarkers. Particularly, multivariate classification approaches for pattern recognition of whole-brain data (i.e., support vector machine, SVM) are becoming increasingly popular in situations in which changes do not affect a single location but are expressed in a distinct pattern across the brain. Using advanced pattern recognition methods, Koutsouleris et al. (2009b) attempted to develop potential MRI-based biomarkers for the prediction of illness outcomes that are reliable and feasible in clinical practice. They convincingly demonstrated extensive GM distribution differences in various categories in individuals at risk for psychosis and morphological pattern differences between individuals at UHR-P and those at UHR-NP. The findings from this study have been replicated in independent populations (Koutsouleris et al., 2012). The expression of progressive dynamic changes in GMV during illness transition has been predicted using neuroanatomical pattern regression (i.e., support vector regression, SVR; Koutsouleris et al., 2010a).

Although the aforementioned studies showed the potential for early recognition and disease prediction in patients at UHR, it is unclear whether MRI-based biomarkers could generalize to clinical practice. There are some unresolved questions regarding the generalization of biomarkers (Kloppel et al., 2012). Given the high classification accuracy from the multivariate pattern recognition approach and the view that schizophrenia is a disorder of large-scale neurocognitive networks rather than specific regions (Menon, 2011), we suggest that neuroanatomical markers for psychosis may be associated with distinct coordinated patterns of cortical morphology rather than with abnormalities in specific regions *per se*. In this sense, the concepts of graph theory (He et al., 2008) and hyper-networks (Ha et al., 2009) may be applied to quantitatively characterize these complex anatomical patterns. Future studies should address issues of clinical and genetic heterogeneity between patients, including differences in symptoms, duration, medication, and genetic vulnerability, which may influence neurobiological substrates in a complex way. The human brain is not static, but dynamically changes throughout life. MRI-biomarkers for psychosis are also not static and may therefore indicate the magnitude of change over time.

## Potential Etiopathological Mechanisms for GM Abnormalities and Therapeutic Implications

The underlying basis of GM abnormalities is currently unknown; however, alterations in brain processes involved in the pathology of psychosis may contribute to the structural abnormalities. Structural brain alterations in the fronto-temporal regions observed in patients with psychosis occur before the onset of full-blown psychosis. These regions are the same regions where lesions in animals result in striatal dopaminergic abnormalities (Flores et al., 1996; Lodge and Grace, 2007; Howes and Kapur, 2009). Striatal hyperdopaminergia has been postulated to be fundamental to the emergence of the psychotic symptoms (Kapur et al., 2005). Recent studies indicate that elevated striatal dopamine function predates the onset of psychosis in UHR subjects (Howes et al., 2009; Fusar-Poli et al., 2010a). It is thus conceived that striatal dopaminergic elevation is present in a compromised brain in psychosis and GM alterations may be associated with dopaminergic subcortical alterations (Howes and Kapur, 2009).

Based on findings from post-mortem studies, GMV alterations may be due to changes in neuronal, synaptic, and dendritic density, as well as increased afferentation in certain regions (Fornito et al., 2009). The combined volume of synapses, axons, and dendrites make up approximately 70% of the total GMV (Chklovskii et al., 2002; Fingerman et al., 2010). Abnormalities in specific region may result from different causes in different individuals (Fornito et al., 2009). Therefore, differences in developmental trajectories may result in significant differences between patients and controls (Fingerman et al., 2010). Recent works by Fornito et al. (2009) have helped to bridge the gap between neuroimaging and the neuropathology of schizophrenia, particularly in the ACC. Differences in cortical GMV may be due to differences in the neuropil (Sowell et al., 2003), where the synaptic connections between branches of axons and dendrites are formed. Therefore, excessive synaptic pruning during adolescence may contribute to the structural changes. However, the effects of synapse loss are less than the differences in GMV observed between patients and controls, as synaptic boutons constitute approximately 10% of GMV (Chklovskii et al., 2002; Fingerman et al., 2010). One-third of the signal in T1-imaged cortex is from white matter (Paus, 2005); therefore, myelination of excess connections (increased afferentation) during adolescence and early adulthood may contribute to the reductions in GMV observed on MRI (Benes et al., 1987). Any alterations in apoptotic mechanisms associated with cell death may also contribute to differences in GMV.

Therefore, it may be valuable to discuss the causes of aberrant apoptotic mechanisms in relation to contemporary theories of psychosis. Note that the following suggestions are purely speculative. One possibility is the involvement of immune and inflammatory mechanisms, which directly affect neuronal proliferation, differentiation, migration, and apoptosis (Ader, 2007). It has been hypothesized that viruses could directly damage brain structures, or a virus may come in contact with the fetal brain and induce a dysfunctional reaction in the immature immune system, leading to autoimmune pathology (Kirch, 1993). Among antibodies against neurotropic viruses, studies have focused on the family of herpes viruses. Exposures to herpes simplex virus type 1 (HSV1) in patients with UHR was associated with structural abnormalities in the cuneus, which is consistent with the region found in HSV1-infected patients with established schizophrenia (Whitford et al., 2012).

Another possibility is the experience of psychological stress around the time of illness onset (Lodge and Grace, 2011). Generally, one biological system proposed as the link between the psychological experience of stress and the development of psychosis is the hypothalamic–pituitary–adrenal (HPA) axis (Phillips et al., 2006). Garner et al. (2005) examined pituitary volume as an indirect measure of HPA-axis dysfunction and found a larger pituitary volume in UHR-P compared with UHR-NP patients. Thompson et al. (2007) found that compared with UHR-NP, UHR-P patients displayed lower plasma cortisol levels at baseline, although there was no correlation between these levels and stressful events, suggesting that HPA-axis dysfunction is involved in the development of psychosis.

Recent studies have suggested the role of stress in GMV alterations in corticostriatal-limbic regions associated with emotional regulation and cognitive control. This is consistent with studies in individuals at UHR, suggesting that these changes may serve to mediate vulnerability to later psychopathology. A significant relationship between cumulative adversity/stress and smaller GMV in the medial PFC, ACC, and insula was found in adults (Ansell et al., 2012). Exposure to childhood maltreatment was associated with reduction in corticostriatal-limbic GM (Edmiston et al., 2011). Moreover, the results of preliminary analyses showed different regional patterns of decreases in GM between males and females; females showed GM reductions in regions associated with emotional regulation, whereas males showed GM reductions in regions involved in impulse control. The authors speculated that vulnerabilities to mental illness in adolescence may be moderated by gender. These sexually dimorphic developmental trajectories are associated with sex hormones and may contribute to sex differences in psychiatric disorders. Pubertal hormones play a role in brain development during the transition from childhood to adolescence, including structural brain-circuit remodeling, myelination, neural pruning, apoptosis, and dendritic spine remodeling (Vigil et al., 2011). Indeed, estrogen has neuroprotective effects on the brain (Norbury et al., 2003). For example, it acts against several toxins that boost the production of free radicals (Simpkins et al., 1994) and may itself act as an antioxidant to protect membranes against reactive oxygen species (ROS) as byproducts of oxygen metabolism (Behl et al., 1995). There is a high consumption of oxygen and enrichment of polyunsaturated fatty acids (PUFAs) in the neuronal membrane. Therefore, the brain is highly susceptible to oxidative damage from free radical attack, called lipid peroxidation. Oxidative stress due to an excess of ROS or a decrease in antioxidant levels results in brain damage and can lead to neurodegenerative diseases. Estrogen may play a role as an antioxidant. Estrogen also modulates the neurochemical transmitter systems, including the serotonergic, cholinergic, noradrenergic, and dopaminergic systems (Norbury et al., 2003). These neurotransmitter systems are associated with the symptoms of schizophrenia. The effects of estrogen on neurotransmitters may partially explain why late-onset schizophrenia is more common in post-menopausal women (i.e., decline in levels of estrogen) and the disorder presents earlier in men than in women. This concept is known as the estrogen-protection hypothesis of schizophrenia (Riecher-Rossler and Kulkarni, 2011). Kulkarni et al. (2012) recently reviewed this hypothesis and suggested that estrogen may offer a novel therapeutic strategy. Alternatively, the testosterone-protection hypothesis states that testosterone protects against schizophrenia by modulating the neurotransmitters implicated in the disorder (Duggal et al., 2002). Testosterone also plays a neuroprotective role in the brain (Hammond et al., 2001), and lower testosterone in patients with schizophrenia was associated with a greater number of negative symptoms (Akhondzadeh et al., 2006). van Rijn et al. (2011) recently investigated levels of salivary testosterone and estradiol in male UHR subjects and found lower testosterone levels and normal estradiol levels in male UHR subjects compared with male controls. This suggests potential neuroendocrine markers for UHR patients. The early effect of testosterone on neurodevelopment in patients with schizophrenia was demonstrated in an abnormal ratio between the lengths of the second and fourth digits (2D:4D ratio; Collinson et al., 2010). The ratio is a marker of the amount of prenatal circulating testosterone and represents early organizational effects of testosterone on brain development (Lutchmaya et al., 2004). Taken together, these findings suggest that gonadal hormones protect against the symptoms of schizophrenia. More than a century ago, Kraepelin first proposed that “dementia praecox” may be due to an imbalance of sexual hormones (Kendler and Jablensky, 2011). Based on previous studies, we suggest that vulnerability to psychosis may reflect an imbalance in sexual hormones rather than a deficiency in a single hormone. Specifically, aberrant sex hormones, particularly during adolescence and early adulthood, may affect structural brain abnormalities.

Structural brain abnormalities in psychosis can be associated with deficits in lipid membrane and essential fatty acids. Previous studies have shown abnormalities in membrane function, including the metabolism and structure of membrane phospholipids involving PUFAs in psychosis. These have been implicated in the membrane hypothesis of schizophrenia (Horrobin et al., 1994; Mossaheb et al., 2012). Specifically, mitochondrial dysfunction due to irregular cellular metabolism and oxidative processes (Clay et al., 2011) and redox/glutathione dysregulation as a result of oxidative stress from dysregulation of glutathione synthesis have been reported (Bokkon and Antal, 2011). The long-chain PUFAs, such as the omega-3 fatty acids, eicosapentaenoic (EPA) and docosahexaenoic (DHA) acids, and the omega-6 fatty acids such as arachidonic acid (AA), are structural components of neuronal membranes. They play a crucial role in brain function as well as normal growth and development including neuronal growth, dendritic arborization, and synapse formation (Kawakita et al., 2006). Thus, consumption of omega-3 PUFAs may influence brain morphology. Long-chain omega-3 fatty acid intake in healthy adults has been associated positively with corticolimbic GMV in the subgenual ACC, hippocampus, and amygdala (Conklin et al., 2007). However, a recent meta-analysis has clearly indicated PUFA treatment are not effective in schizophrenia (Fusar-Poli and Berger, 2012). Conversely, in UHR patients, a 12-week intervention with omega-3 significantly reduced the transition rate to psychosis (22.6%) compared with the placebo group. Moreover, the treatment resulted in significant symptomatic and functional improvements during the entire 12-month follow-up period (Amminger et al., 2010). Future studies should investigate the relationship between omega-3 PUFAs and structural GMV with respect to preventing the onset of psychosis in UHR patients.

The contribution of membrane turnover in the prediction of psychosis may also involve using 31P-magnetic resonance spectroscopy (MRS) to measure the phosphomonoesters and phosphodiesters levels that are reflective of membrane phospholipid precursors and breakdown products (Reddy and Keshavan, 2003). The relationship between cerebral morphology and membrane phospholipid metabolism, as measured by phosphorus-MRS (31P-MRS), was reported in patients with schizophrenia (Keshavan et al., 1993). Patients with schizophrenia showed altered metabolism of membrane phospholipids during the early course of the illness, which is consistent with a neurodevelopmental abnormality around the critical period of adolescence (Stanley, 2002). Proton-MRS (1H-MRS) has also been used to measure metabolite concentrations, such as choline, creatine, N-acetylaspartate (NAA), glutamine, and glutamate in the brain. In patients at UHR, 1H-MRS studies showed normal levels of NAA, a marker of neuronal/axonal integrity, in the hippocampus of UHR subjects (Wood et al., 2003, 2010). UHR subjects had metabolic changes in the PFC and ACC that were investigated using NAA/creatine and NAA/choline ratios (Wood et al., 2003; Byun et al., 2009). Based on growing evidence for the involvement of glutamatergic abnormalities in schizophrenia, 1H-MRS studies in patients with UHR investigated glutamate levels in the brain. Glutamatergic dysfunction may have an impact on dopaminergic transmission and ultimately lead to the onset of psychosis. Studies have shown an altered relationship between glutamate levels and striatal dopamine function in UHR patients (Stone et al., 2010). Multimodal studies combining MRI and MRS in UHR subjects indicated that glutamatergic dysfunction in the thalamus is associated with a reduction in GMV in the medial temporal cortex and insula (Stone et al., 2009). Thus, imbalanced neurochemicals may underlie structural brain abnormalities in psychosis.

Structural GM abnormalities may be associated with *N*-methyl-d-aspartate (NMDA) receptor hypofunction, termed the excitotoxic hypothesis of psychosis-induced apoptosis (Farber, 2003). According to this hypothesis, disinhibited excitation of NMDA receptors on gamma-aminobutyric acid (GABA)-ergic interneurons results in excessive stimulation of the class of glutamate receptors and excitotoxicity, including the generation of ROS and apoptotic cell death in hippocampal and cortical areas (Farber, 2003). The involvement of NMDA receptor hypofunction in schizophrenic pathophysiology has been supported by findings that non-competitive NMDA receptor antagonists, phencyclidine (PCP), or ketamine, produce schizophrenia-like symptoms in healthy individuals and profoundly exacerbate pre-existing symptoms in patients with psychosis.

Taken together, these findings suggest that excessive oxidative stress and deficits in the ability to cope with oxidative stress as a result of genetic, epigenetic, and environmental risk factors may result in structural GM abnormalities. Thus, neuroprotective strategies may be used as potential treatments to delay or prevent the progression of the disorder in UHR patients and halt progressive atrophy in patients with psychosis. In this regard, the use of recombinant human erythropoietin (EPO), a neuroprotective hormone (Brines and Cerami, 2005), may improve neuroplasticity (Adamcio et al., 2008) and delay the loss of GMV (Wüstenberg et al., 2011). More recently, low-dose lithium has been shown to have neuroprotective effects on hippocampal microstructure in a small UHR group in a longitudinal MRI–MRS study (Berger et al., 2012).

Considering ethical issues related to drug intervention in subjects at UHR, an interesting option is to target nutrition. Nutrition affects the brain throughout life (Bourre, 2006a,b; Dauncey, 2009). Early nutrition affects brain structures and cognitive function in later life (Isaacs et al., 2008). A recent VBM study investigated whether breakfast foods (i.e., bread vs. rice) affect GMV and cognitive function in healthy children (Taki et al., 2010). The rice group showed higher IQ scores and greater GMV in the STG, inferior frontal gyrus, and caudate nuclei, whereas the bread group displayed greater GMV in the fronto-parietal region. The authors speculated that a possible mechanism might be the difference in the glycemic index (GI) between the two groups; low GI foods are associated with less blood-glucose fluctuation compared with high GI foods.

Long-chain PUFAs and antioxidants protect neurons against oxidative stress. Antioxidants such as vitamins E, C, and A (beta-carotene), glutathione, and minerals cannot be synthesized *de novo* in humans and are therefore required from dietary sources. PUFAs play a crucial role in brain functioning, as well as in normal growth and development. It is important to consider the ratio of omega-6/omega-3 fatty acids in the diet (Simopoulos, 2002) as well as the consumption of nutrients that can influence omega-3 status (Berr et al., 2009). Excessive levels of omega-6 promote the pathogenesis of many diseases, including inflammatory and autoimmune diseases, whereas increased levels of omega-3 PUFAs exert suppressive effects (Simopoulos, 2002). Thus, we suggest that nutritional balance and nutrient interactions are important for mental health and brain development. Further studies should focus on nutritional balance in UHR patients and its relationship with GMV.

## Potential Issues and Future Directions

Recent advances in psychiatric research have focused on early detection and prevention of psychosis (McGorry et al., 2009). However, MRI-based biomarkers for UHR patients should be targeted to improve the specificity and sensitivity of criteria that are presently available. Although there have been attempts, to date, the use of MRI-based biomarkers for individuals with psychosis in a clinical setting has not been successful.

First, methodological variability may be a source of heterogeneity across studies (Fusar-Poli et al., 2008a). For example, different scanners (i.e., 1.5 or 3 T), different methods of data analysis, and differences in imaging parameters need to be addressed (Fusar-Poli et al., 2010b). Because a small sample size limits the power to detect differences, there is a growing probability that the results of some studies include false positives. Therefore, multicenter studies are needed to investigate the key comparison between UHR-P and UHR-NP.

Second, antipsychotic exposure is a confounding factor. Previous studies have indicated that antipsychotic treatment in UHR patients may alter GMV in the frontal and temporal areas (Smieskova et al., 2009). Atypical antipsychotic treatment may reverse such alterations (i.e., increased GMV in the inferior frontal gyrus) and prevent the onset of psychosis (Fusar-Poli et al., 2011b). Although recent neuroimaging studies have taken into account the effects of antipsychotics, their effects on the brain cannot be ignored when investigating patients with chronic schizophrenia. Chronic patients generally use antipsychotics. Specifically, the effects of medication exposure should be taken into consideration in studies of patients at UHR-P and UHR-NP.

Third, the criteria used to identify UHR subjects differ across studies. The majority of studies use the Comprehensive Assessment of Symptoms and History (CAARMS; Yung et al., 2005) or the newly developed and shorter Basel Screening Instrument for Psychosis (BSIP; Riecher-Rössler et al., 2007). Other employs the Structured Interview for Prodromal Symptoms (SIPS)/the Scale of Prodromal Symptoms (SOPS) or the basic symptoms (BS) approach. Discrepancies in previous findings may result from differences in trait/state risk factors and BS among UHR individuals for psychosis. However in the recent meta-analysis including over than 2550 HR subjects, the transition risk was comparable across different inclusion criteria (Fusar-Poli et al., 2012b). A further problem is that the UHR group is heterogeneous and typically include both UHR-P and UHR-NP subjects, as well as patients at different levels of risk for psychosis. UHR groups can be divided into two groups based on their symptoms or on the duration of the ARMS status since first presentation. Such divisions might sort patients, respectively, as (i) individuals in the early prodromal state (EPS) with trait vulnerability based on their symptoms and (ii) individuals in the late prodromal state (LPS) with APS and BLIPS (Häfner et al., 1992) or as (i) individuals with short-term ARMS status (ARMS-ST) and (ii) those with long-term ARMS status (ARMS-LT) based on the time since first presentation (McGorry et al., 2009; Smieskova et al., 2012a,b). APS and BLIPS subjects are closer to transition to psychosis than are subjects in the EPS, including trait subjects. Indeed, the intake criteria for UHR subjects predicted transition over 6 months in the order of trait alone <APS <BLIPS (Nelson et al., 2011). Hurlemann et al. (2008) reported that although both EPS and LPS patients had reduced hippocampal GMV, the reductions were correlated with poorer cognitive performance in only the LPS. Koutsouleris et al. (2009a) also investigated differences in GMV between EPS and LPS patients and between UHR-P and UHR-NP groups. They found PFC alterations in UHR-P relative to UHR-NP and controls, which overlapped with the findings in LPS. Jung et al. (2011) found significantly reduced cortical thickness in the STG, PFC, ACC, and parietal area of the group with APS and BLIPS compared with healthy controls. Koutsouleris et al. (2009b) recently distinguished between EPS and LPS using GMV differences between groups with multivariate pattern classification.

A previous study that investigated the differences between ARMS-ST (higher probability) and ARMS-LT (lower probability) improved our understanding of early intervention in the context of a clinical staging model (McGorry et al., 2009). ARMS-ST displayed less GMV in the insula compared with ARMS-LT and FEP. Moreover, these GMV differences were correlated with symptoms (Smieskova et al., 2012b). A multimodal fMRI-VBM approach revealed smaller GMV in the temporal gyrus and extending into the insula in ARMS-ST compared with ARMS-LT. Moreover, a correlation was noted between the reductions in GM and reduced activation in the same area of ARMS-LT (Smieskova et al., 2012c). The authors speculated that the differences between ARMS-ST and ARMS-LT was related to increased vulnerability-associated alterations in ARMS-ST and to resilience or protective processes in ARMS-LT. Further studies should take into account different levels of risk for psychosis to clarify vulnerability-related, disease-related, and resilience biomarkers. It would be interesting to investigate cortical changes associated with the duration of the ARMS status after first presentation in terms of a progressive neurodevelopmental model of schizophrenia. However, that study must note whether patients identified as ARMS-LT were originally non-psychotic (i.e., healthy subjects with false-positive assessment) or remained non-psychotic through their resilience or protective processes.

Fourth, subjects who meet UHR criteria can transition to schizophrenia as well as to other psychiatric disorders including bipolar disorder. This prevents the development of disease-specific prevention strategies and limits the clinical applicability of ongoing basic research (Fusar-Poli et al., 2008b). The criteria are able to identify those at UHR for psychosis but not for schizophrenia. Separate criteria exist for the diagnosis of bipolar disorder (BAR; Bechdolf et al., 2012a). Previous MRI findings in UHR subjects are related to psychosis rather than to schizophrenia. Recent studies have attempted to distinguish UHR subjects who converted to a diagnosis of schizophrenia (UHR-SZ) from other UHR subjects who converted to other disorders. Dazzan et al. (2012) divided UHR subjects into three groups; UHR-SZ, UHR who converted to affective psychosis (UHR-AFF), and UHR-NP. Decreased volume in the parietal cortex and a trend toward decreases in the middle temporal and inferior frontal cortices were noted in UHR-SZ subjects, and smaller superior frontal volumes were noted in UHR-AFF compared with UHR-NP subjects. Additionally, reduced subcallosal cingulate volume was noted in UHR-AFF subjects compared with UHR-SZ subjects. Bechdolf et al. (2012b) investigated amygdalar, insular, lateral ventricular, and whole-brain volumes in subjects at UHR, all of whom had developed bipolar I or II disorder (UHR-BP) at the time of a follow-up session, compared with UHR-NP and healthy controls. They found reduced amygdalar and insular volumes in UHR-BP subjects compared with UHR-NP subjects and controls. An approach that distinguishes UHR-SZ subjects from individuals with other psychosis will enable the development of specific biomarkers to detect and prevent the onset of schizophrenia. Moreover, a thorough understanding of the neurobiological nature of psychiatric diseases, along with knowledge of common and different structural abnormalities before and after the onset of different psychotic disorders, is warranted.

Fifth, it is still unclear to what extent state and genetic factors influence structural brain alterations in patients with psychosis and, particularly, what shared genetic factors in UHR patients influence their structural abnormalities. This information will need to be clarified to improve the feasibility of MRI-based biomarkers. The findings from previous studies that compared EPS with LPS may implicate more state-related abnormalities than do other UHR studies. Few studies have directly compared UHR and GHR. A recent meta-analysis of VBM studies with GHR and antipsychotic-naïve FEP revealed reduced GMV in the parahippocampal gyrus and ACC in GHR compared with controls and decreased volumes in the ACC, precuneus, cerebellum, and STG in FEP compared with GHR (Fusar-Poli et al., 2012e). The authors concluded that reductions in GM in the ACC are markers of genetic liability to psychosis, whereas reductions in the STG and cerebellum are markers of the onset of the illness. Another voxel-wise meta-analysis was conducted on VBM studies of subjects at UHR and GHR (Fusar-Poli et al., 2011b). Compared with controls, reductions in GM were observed in the parahippocampal gyrus, STG, insula, and inferior and medial frontal gyri in UHR subjects and in the parahippocampal gyrus and ACC in GHR subjects. Direct comparisons between subjects at UHR and GHR showed that subjects at UHR had less GMV in the ACC and greater GMV in the parahippocampal gyrus, insula, and STG than did GHR subjects.

Sixth, there are only a few longitudinal studies on this topic. Adolescence is a critical period in the development of the human brain, which is particularly vulnerable to the onset of psychosis at this time (Gogtay et al., 2011). Developmental changes in GMV occur at different rates in different brain regions (Sowell et al., 2003; Gogtay et al., 2004). Pantelis et al. (2010) focused on progressive changes in GM in the PFC, insula, and STG throughout the transition to psychosis in longitudinal studies of patients with UHR, as opposed to inconsistent findings from cross-sectional comparisons in the same cohorts. Trajectories of brain development may be more informative than cross-sectional studies of structural abnormalities. These studies suggested acceleration of the normal maturational process in vulnerability to psychosis (Sun et al., 2009a; Gogtay et al., 2011). Many researchers have suggested the heritability of dynamic brain changes in normal individuals and non-affected relatives (Gogtay et al., 2007; Raznahan et al., 2011). If abnormalities in normal development are potential endophenotypes, the concept of the endophenotype in schizophrenia should be redefined (Pantelis et al., 2010). Future studies should clarify the theory that genes associated with brain development during adolescence and early adulthood affect brain abnormalities by accelerating the process of normal neurodevelopment.

Seventh, the effect of sex on brain abnormalities in subjects at UHR remains unclear. The prevalence of psychosis is greater in males throughout most of adulthood, but is equal in males and females by the end of the risk period (MacDonald and Schulz, 2009). Furthermore, sex hormones influence the onset of psychosis and the associated brain abnormalities.

Eighth, a variety of structural MRI studies in subjects at UHR now exist, including single MRI and multi-imaging modalities. Multiple papers have been published on patients at UHR but it is unclear how many subjects overlap in the published studies. Further meta-studies should take into account the direct overlap between subjects.

Finally, the relationship between structural brain abnormalities and cognitive dysfunction during the transition to psychosis is unclear. One recent study investigated the relationship between structure and cognition in UHR patients (Koutsouleris et al., 2010b). Whereas a relationship between executive function and insular GMV was noted in healthy controls, impaired executive function and positive correlations with the cerebellum and negative correlations with the rectus were noted in UHR subjects. A relationship between executive function and caudate volume in UHR subjects has recently been reported (Hannan et al., 2010).

Taken together, these observations indicate that additional longitudinal multimodal studies with larger samples of drug-naïve subjects who convert to different psychoses, conducted across numerous centers and with data from multiple scanners are necessary to address the aforementioned issues. Additionally, the investigation of different features of cortical areas, including structural asymmetries, gyrification, and shape will provide optimal MRI-based biomarkers for psychosis.

## Conclusion

Over the past generation, MRI studies in prodromal psychosis have exposed a “smoking gun” with respect to whether there are brain abnormalities and what brain regions are involved in the pathophysiology of psychosis. Indeed, MRI studies represent a marvelous approach that provides new knowledge about the neurobiological trajectories of the disorder. Structural brain abnormalities in psychosis occur prior to full-blown symptoms and progressively worsen over the course of the illness. The discoveries from MRI studies, in combination with evidence from genetic, neurochemical, and environmental studies, are prompting a paradigm shift in psychosis research. These data are contributing to a progressive neurodevelopmental model, i.e., a combined neurodevelopmental and neurodegenerative model (Rapoport and Gogtay, 2011). Furthermore, MRI studies can confirm the safety evaluation and effectiveness of new candidate drugs and non-drug interventions. The identification of regions associated with the onset of psychosis may localize targets for treatment interventions. Recent advances in clinical and neuroimaging techniques suggest the feasibility of MRI-based biomarkers for early interventions. Knowledge of metabolic processes that regulate homeostatic balance in the brain can greatly improve the development of new drugs that protect against structural brain abnormalities. All of the hypotheses regarding brain abnormalities associated with the onset of psychosis may converge into the idea that abnormalities in specific brain processes of energy metabolism are affected by genetic and/or environmental factors. In the context of impaired brain energy metabolism, brain abnormalities and psychosis may result from an imbalance of neurochemical factors and nutritional status. In this regard, neuroprotective agents can interrupt structural brain alterations and symptoms of psychosis. Moreover, nutritional components may affect neuroplasticity. Psychosis vulnerability studies that use neuroimaging techniques will help to bridge the gap between fundamental knowledge of brain abnormalities in psychosis and therapeutic implications for treatment and hence pave the way for new treatment strategies.

## Conflict of Interest Statement

The authors declare that the research was conducted in the absence of any commercial or financial relationships that could be construed as a potential conflict of interest.
